# Supplementary material for: Plasmodium transcription repressor AP2‐O3 regulates sex‐specific identity of gene expression in female gametocytes
Source: EMBO Rep. 2021 Mar 4;22(5):e51660. doi: 10.15252/embr.202051660 (PMC8097350; doi:10.15252/embr.202051660)
Supplement: Supplementary file 1 — Appendix [file EMBR-22-e51660-s003.pdf]

Appendix

***Plasmodium* transcription repressor AP2-O3 regulates sex-specific identity of gene expression in female gametocytes**

Zhenkui Li[1,†], Huiting Cui[1,†], Jiepeng Guan[1], Cong Liu[1], Zhengang Yang[1], and Jing Yuan[1,*]

[1] State Key Laboratory of Cellular Stress Biology, Innovation Center for Cell Signal Network, School of Life Sciences, Xiamen University, Xiamen, Fujian 361102, China

* Corresponding author. E-mail: yuanjing@xmu.edu.cn

[†] These authors contributed equally to this work.

Table of Contents

Page 2-3: Appendix Figure S1 and figure legend.

Page 4-5: Appendix Figure S2 and figure legend.

Page 6-7: Appendix Figure S3 and figure legend.

Page 8-9: Appendix Figure S4 and figure legend.

Page 10-11: Appendix Figure S5 and figure legend.

Page 12: Appendix Table S1-List of genetically modified parasite strains in this study.

Page 13: Appendix Table S2-Primers and oligonucleotides used in this study.

Appendix Figure S1

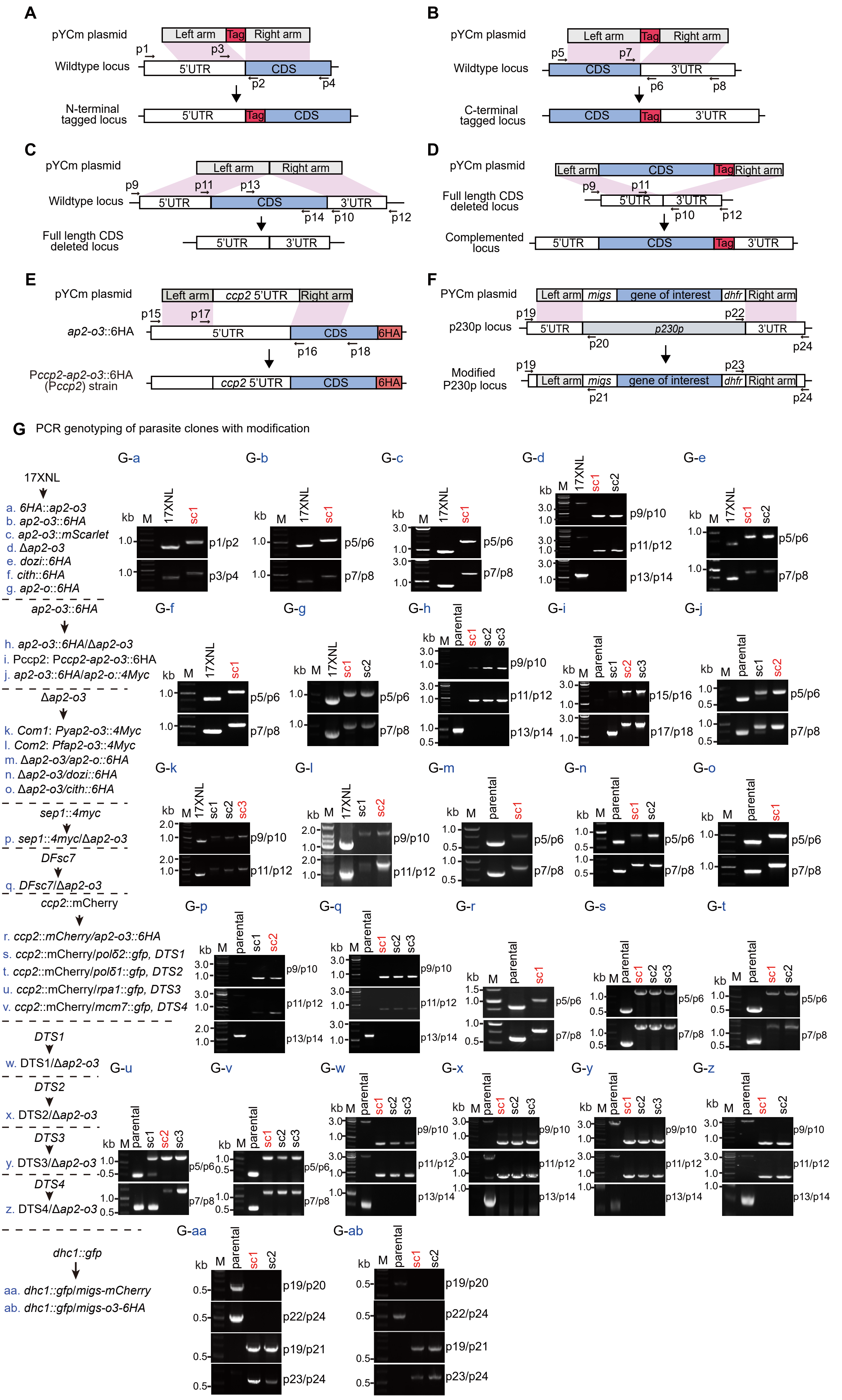

Appendix Figure S1. Genotyping of modified parasites.

A-F Schematic of CRISPR/Cas9-mediated gene modification via double cross homologous recombination, including gene tagging in the N-terminus (A) and C-terminus (B), deletion of full-length coding sequence (CDS) (C), gene complementation (D), promoter replacement (E) and ectopic expression (F).

G For each modification, both 5' and 3' homologous recombination were detected using gene specific PCR pair (Appendix Table S2) to confirm correct integration of the homologous template. Usually, one to three parasite clones (sc) for each modification were obtained after limiting dilution, and the clone indicated with red letter is used for phenotype and functional analysis.

Appendix Figure S2

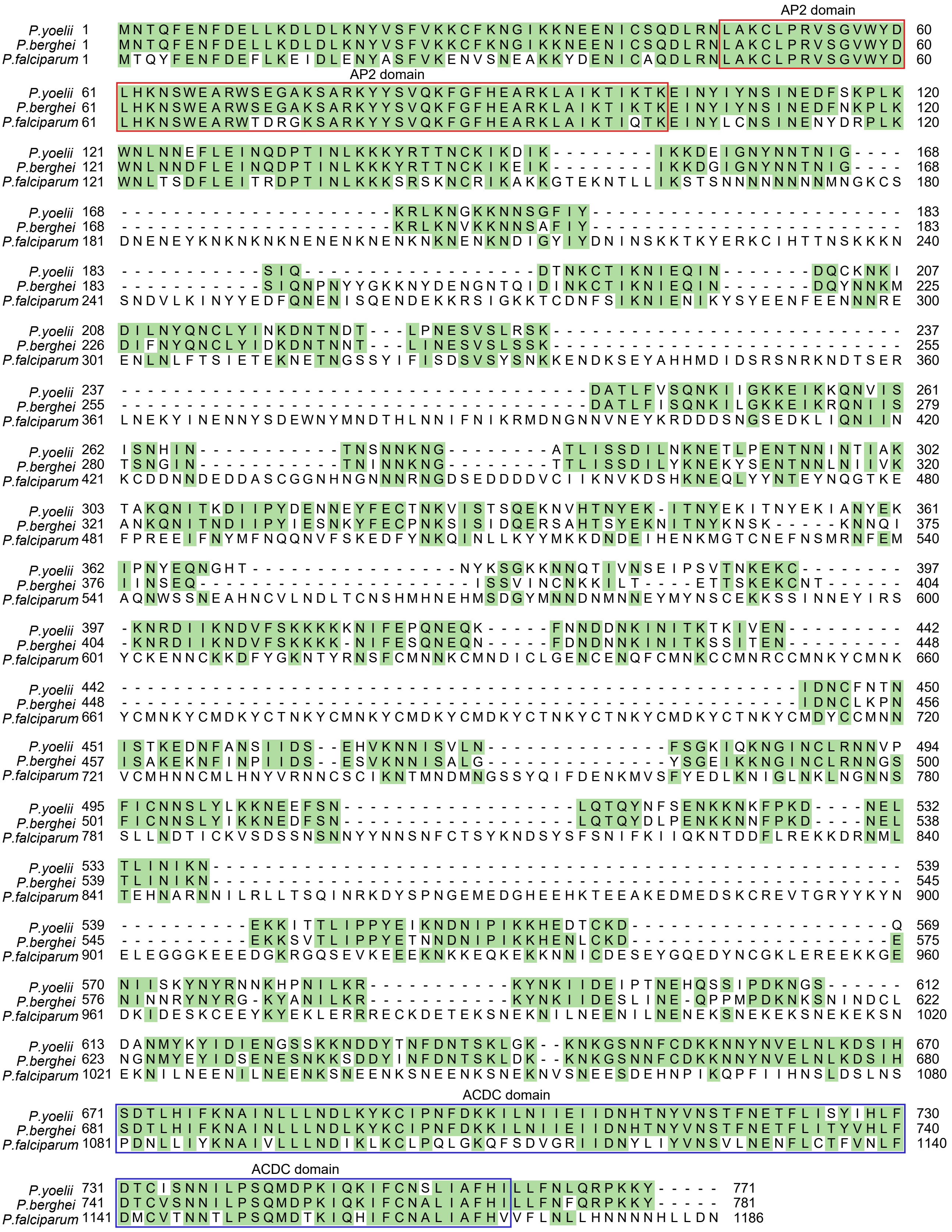

Appendix Figure S2. Protein sequence alignment of AP2-O3 among *Plasmodium* parasites.

Aligned AP2-O3 protein amino acid sequences from *P. falciparum* (PF3D7_1429200), *P. berghei* (PBANKA_1015500), and *P. yoelii* (PY17X_1017000). The AP2 (DNA binding domain) and ACDC domains are indicated with red and blue lines respectively.

Appendix Figure S3

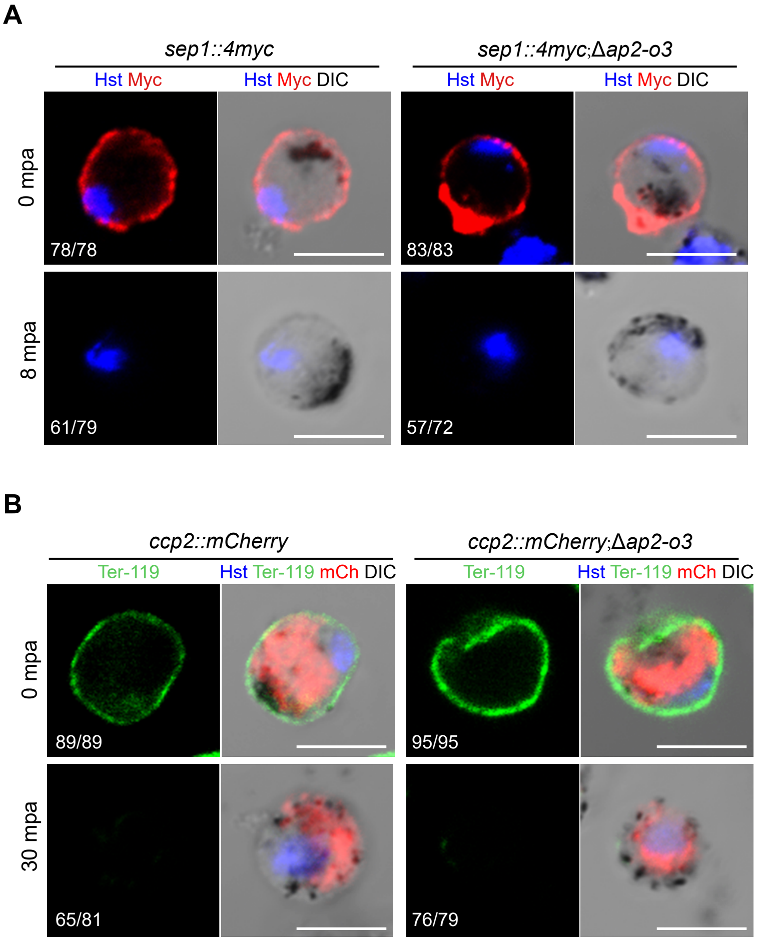

Appendix Figure S3. AP2-O3 is dispensable in PVM and EM rupture during gametogenesis.

A Representative IFA images of the *sep1::4Myc* and *sep1::4Myc;*Δ*ap2-o3* gametocytes stained by anti-Myc antibody, showing the disappearance of PVM (Myc tagged SEP1) in the *sep1::4Myc* and *sep1::4Myc;*Δ*ap2-o3* infected RBC 0 and 8 min post after XA stimulation.

B Representative IFA images of *ccp2::mCherry* and *ccp2::mCherry;*Δ*ap2-o3* gametocytes stained by anti-mCherry and anti-mouse TER119 antibodies 0 and 30 min post XA stimulation. mCherry protein expression indicates female gametocyte.

Data information: x/y in the figure is the number of a cell displaying signal/the number of cells tested. Scale bars = 5 μm for all images.

Appendix Figure S4

A

Gene ID	Gene name	Mean±SD FPKM in *DFsc7*	Mean±SD FPKM in *DFsc7;Δap2-o3*	p-value	Log2(FC)
PY17X_1220900	*dozi*	289.9±45.1	401.8±20.9	0.0175	0.47
PY17X_1304900	*cith*	188.1±19.2	268±32.5	0.0215	0.51
PY17X_0415700	*eIF4e*	46±4.9	75.4±3.6	0.0011	0.71
PY17X_1441700	*pabp1*	429.8±62.6	698.3±22.1	0.0022	0.70
PY17X_1035100	*celf2*	61±6.9	88.3±3.9	0.0039	0.53
PY17X_1425300	*alba1*	1478.8±190.3	1970.1±95.2	0.0161	0.41
PY17X_1364900	*alba2*	168.9±7.7	254.8±17.3	0.0014	0.59
PY17X_1207600	*alba3*	134.2±28.3	215.5±16.6	0.0127	0.68
PY17X_0410900	*phosphoglycerate mutase*	44.9±4.4	55.7±6.6	0.0791	0.30

B

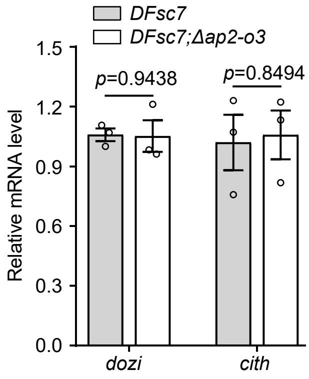

C

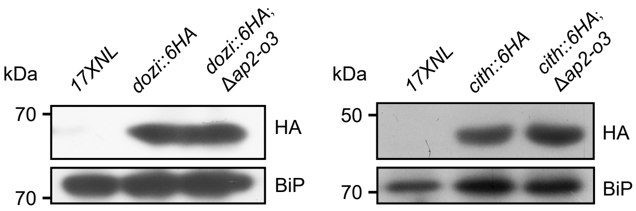

D

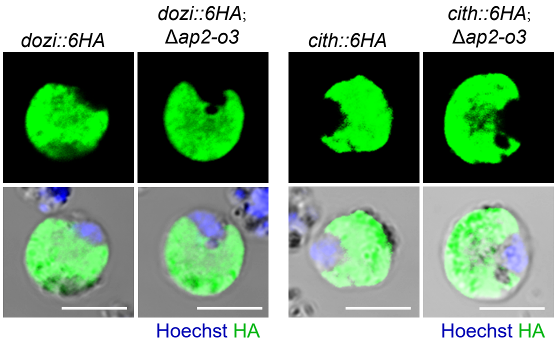

Appendix Figure S4. Transcriptions of genes participating in the translational repression are not decreased in *ap2-o3* null female gametocytes.

A List of nine genes encoding components of translational repression complex in *Plasmodium* female gametocytes. Transcript level (FPKM value) of these genes by RNA-seq was indicated in female gametocytes of the *DFsc7* (WT) and *DFsc7/*Δ*ap2-o3* (KO) strains.

B qRT-PCR of transcript level of *dozi* and *cith* genes, two essential component factors of translational repression complex. mean±SEM from three independent experiments. Two-tailed unpaired Student's t-test applied.

C,D Expression of DOZI protein in non-activated gametocytes of the tagged strains *dozi::6HA* and *dozi::6HA*; Δ*ap2-o3* and CITH protein in non-activated gametocytes of the tagged strains *cith::6HA* and *cith::6HA*; Δ*ap2-o3* via western blot (C) and IFA (D). BiP as loading control. Scale bars = 5 µm. All experiments were repeated three times independently.

Appendix Figure S5

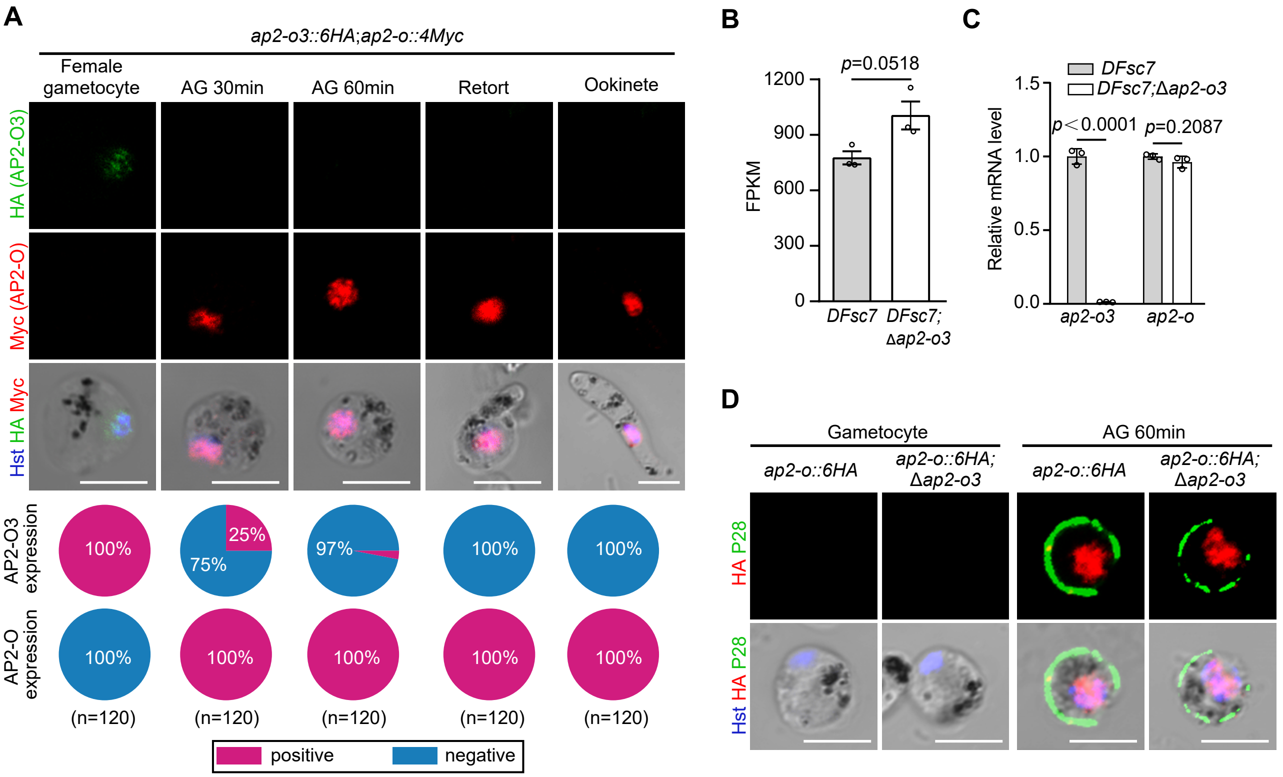

Appendix Figure S5. AP2-O3 and AP2-O exhibit mutual exclusive protein expression from gametocyte differentiate into ookinete.

A Time course IFA analysis of both AP2-O3 and AP2-O proteins during female gametocyte to ookinete development of the double-tagged strain *ap2-o3::6HA;ap2-o::4Myc*. Scale bars = 5 μm. Pie charts exhibit the percentage of cells with or without protein expression. "n" is the number of cells counted.

B Transcript level (FPKM value) of the *ap2-o* gene by RNA-seq in female gametocytes of *DFsc7* (WT) and *DFsc7;*Δ*ap2-o3* (KO) strains. mean±SEM from three repeats. Two-tailed unpaired Student's t-test applied.

C qRT-PCR of transcript level of *ap2-o* gene. mean±SEM from three experiments. Two-tailed unpaired Student's t-test applied.

D IFA of AP2-O and P28 proteins expression in non-activated and activated gametocytes of the *ap2-o::6HA* and *ap2-o::6HA;*Δ*ap2-o3* strains. Scale bars = 5 μm for all images. The data are representative of three independent experiments.

Appendix Table S1. List of genetically modified parasite strains in this study.

Strain	Parental strain	Description	Resource
17XNL	/	*Plasmodium yoelii*	NIH
**Parasites with gene tagging**			
6HA::ap2-o3	17XNL	*Pyap2-o3* N-terminally tagged with 6HA	in this study
ap2-o3::6HA	17XNL	*Pyap2-o3* C-terminally tagged with 6HA	in our lab
ap2-o3::mScarlet	17XNL	*Pyap2-o3* C-terminally tagged with mScarlet	in this study
dozi::6HA	17XNL	*Pydozi* C-terminally tagged with 6HA	in this study
cith::6HA	17XNL	*Pycith* C-terminally tagged with 6HA	in this study
ap2-o::6HA	17XNL	*Pyap2-o* C-terminally tagged with 6HA	in this study
dozi::6HA	*Δap2-o3*	*Pydozi* C-terminally tagged with 6HA	in this study
cith::6HA	*Δap2-o3*	*Pycith* C-terminally tagged with 6HA	in this study
ap2-o::6HA	*Δap2-o3*	*Pyap2-o* C-terminally tagged with 6HA	in this study
ap2-o::4Myc	*ap2-o3::6HA*	*Pyap2-o* C-terminally tagged with 4Myc	in this study
ap2-o3::6HA	*ccp2::mCherry*	*Pyap2-o3* C-terminally tagged with 6HA	in this study
dpod2::gfp	*ccp2::mCherry*	*Pydpod2* C-terminally tagged with gfp	in this study
dpod1::gfp	*ccp2::mCherry*	*Pydpod1* C-terminally tagged with gfp	in this study
rpa1::gfp	*ccp2::mCherry*	*Pyrpa1* C-terminally tagged with gfp	in this study
mcm7::gfp	*ccp2::mCherry*	*Pymcm7* C-terminally tagged with gfp	in this study
**Parasites with gene knockout**			
Δ ap2-o3	17XNL	Deleted the whole coding sequences of *Pyap2-o3*	in our lab
Δ ap2-o3	*ap2-o3::6HA*	Deleted the whole coding sequences of *Pyap2-o3*	in this study
Δ ap2-o3	*sep1::4Myc*	Deleted the whole coding sequences of *Pyap2-o3*	in this study
Δ ap2-o3	DFsc7	Deleted the whole coding sequences of *Pyap2-o3*	in this study
Δ ap2-o3	DTS1	Deleted the whole coding sequences of *Pyap2-o3*	in this study
Δ ap2-o3	DTS2	Deleted the whole coding sequences of *Pyap2-o3*	in this study
Δ ap2-o3	DTS3	Deleted the whole coding sequences of *Pyap2-o3*	in this study
Δ ap2-o3	DTS4	Deleted the whole coding sequences of *Pyap2-o3*	in this study
Δmap2	17XNL	Deleted partial coding sequences of *Pymap2*	in our lab
Δnek4	17XNL	Deleted partial coding sequences of *Pynek4*	in our lab
**Other gene modification**			
Δap2-o3/Pyap2-o3::4Myc	*Δap2-o3*	Complemtation with of *P.yoelii ap2-o3*	in this study
Δap2-o3/Pfap2-o3::4Myc	*Δap2-o3*	Complemtation with of *P.falciparum ap2-o3*	in this study
Pccp2-o3-6HA	*ap2-o3::6HA*	Replacment of ap2-o3 promoter with the ccp2 promoter	in this study
Δp230p/migs-mCherry (EE1)	*dhc1::gfp*	Ectopic expression of *mCherry* in *p230p* locus	in this study
Δp230p/migs-ap2-o3 (EE2)	*dhc1::gfp*	Ectopic expression of *ap2-o3* in *p230p* locus	in this study

Appendix Table S2. Primers and oligonucleotides used in this study.

Oligo sequences for gene tagging plasmid construction

Gene name	Tag	Parental strain	Left homologous arm		Right homologous arm		Target site of sgRNA	
			Forward primer	Rerverse primer	Forward primer	Reverse primer	Oligo (Forward)	Oligo (Reverse)
ap2-o3	C-terminal 6HA	17XNL	CCCGGTACCCATGAAGATACATGTAAAGATC	GGGCCATGGATATTTTTTTGGTCGCTGT	GGGCTCGAGTGTCTCCAATGATGGCTGAC	GGGCTTAAGACACATTCACATGTGATTAT	TATTATTTTTGGGTCCATCTGACT	AAACAGTCAGATGGACCCAAAAAT
ap2-o3	C-terminal mScarlet							
ap2-o3	N-terminal 6HA		CCCGGTACCCTCTATACCGTTCCCAATAG	GGGCCATGGTCTTTGAAGCGGTGTAAAAAATAGG	GGGCTCGAGATGAATACACAATTTGAAAAT	CCCGAATTCCATTGATCATTTATTTGTTCG	TATTGTGGAATTTCATGTTAAATG	AAACCATTTAACATGAAATTCCAC
dozi	C-terminal 6HA	17XNL and Δ*ap2-o3*	GCCAAGCTTCCAGCCTATAGTAGAAGAAT	GGGCCATGGAGTATAGATGGATGGATCTA	GGGCTCGAGTTTGCAGTATACTACATCATA	GGGCTTAAGATTCCATAACGTCGAATTTC	TATTGCACATATATATACACATTG	AAACCAATGTGTATATATATGTGC
cith	C-terminal 6HA	17XNL and Δ*ap2-o3*	GCCAAGCTTGATACCTGATGATCCAGCAAT	GGGCCATGGATAGGCTGGATATCTGTTAAATGG	GGGCTCGAGTATATATATATTATCTACTAATTC	GGGCTTAAGGTAAAGTGTGTAACATATTC	TATTTATATGTATTATTTTTCGCT	AAACAGCGAAAAATAATACATATA
ap2-o	C-terminal 6HA	17XNL and Δ*ap2-o3*	GGGAAGCTTGGGTTCTAACTTAGTAGACA	GGGCCATGGGATGTTATCAACAGTAATAC	GGGCTCGAGTAATGAACCGTTTGTATCCA	GGGCTTAAGCCCCAGTATTAAACTCTTTT	TATTGATGGTAAGTGTGGAATGGCT	AAACAGCCATTCCACACTTACCATC
ap2-o	C-terminal 4Myc	*ap2-o3::6HA*						
polδ1	C-terminal GFP	*ccp2::mCherry*	CCCGGTACCGAGCTGAAGATCCATTATATG	CCCGGATCCGCTCCCGCCGGACCATTCAATTTTGAGGGATG	CGGGAATTCAAAGAATAAAAATAACAAGAGGG	GGGCTTAAGTTCTCCTTCTGCGGTAAATT	TATTGGGATGTAACTTGTTCTTGA	AAACTCAAGAACAAGTTACATCCC
polδ2	C-terminal GFP		cccaagcttATGCCAGGAGAAAAAGATCC	cccGGATCCGCTCCCGCCGGATTCGCCTTCTTGAATATTGA	cccGAATTCACTATATCCCTATTTGCATA	gggcttaagAACATTTTGTGAGCATGCTA	TATTGTAAGAAGGTACACATAATTG	AAACCAATTATGTGTACCTTCTTAC
mcm7	C-terminal GFP		CCCAAGCTTCGAAGAAGCTATTCGTTTAACAG	CACGGATCCAAAATCATTTTCTTCATCATCACC	CCCGAATTCTTTCAAATAAAATCAAAATACAAAAATGTG	GGGCTTAAGCACATAACTATCGTGTTTATTCG	TATTATTAACAAAGGGATTTACAA	AAACTTGTAAATCCCTTTGTTAAT
rpa1	C-terminal GFP		cccaagcttGAGCTTTTTCTGATGAAGGG	cccGGATCCTATTTTTTGTTTTTTTGCATC	cccGAATTCAAATTAATTTTTTTAATCGTTAAAATGG	cggCttaagGACGGTTTAAAAAAAATAGTTC	TATTATCATAAGAATTGTCGTTCA	AAACTGAACGACAATTCTTATGAT

Primers of diagnositc PCR for gene tagging

Gene name	Tag	parental strain	P1	P2	P3	P4		
ap2-o3	N-terminal 6HA	17XNL	GTAAAAAGGAAAATTAATTC	CTAGATCTTTTAATAGTTCA	GTATAGTATAGTATAGTAAAAAGCG	GATTCATTTGGTAATGTATC		
**Gene name**	**Tag**	**parental strain**	**P5**	**P6**	**P7**	**P8**		
ap2-o3	C-terminal 6HA	17XNL	CAACTTTGATTCCTCCATATG	CGTGGATCTTTCGCACGAGC	GTCAGATGGACCCAAAAATTC	ATACACACACGCGCACATTTTA		
ap2-o3	C-terminal mScarlet							
dozi	C-terminal 6HA	17XNL and Δ*ap2-o3*	GATGTTGCAAATTTATCAGG	GTATATGTGGGGTAAGTTGG	GAATTAGAACTAGGAACTGA	GTGTGTGACTACGAGCAAAAAG		
cith	C-terminal 6HA	17XNL and Δap2-o3	GTTACAGTAAGTGAAGCCGC	GTAATTCAATATTTGTATATAG	GGACTAATATGCATAATAGAAA	GCTTACTCCGAAAACACATTAT		
ap2-o	C-terminal 6HA	17XNL and Δ*ap2-o3*	GAATCTTGCATCTAACAATA	CCATCACACTTCACGCTTCT	AGGGCAAACTATTTTTGGAA	AAGAACAAGTTGTTTCTTCG		
ap2-o	C-terminal 4Myc	*ap2-o3::6HA*						
polδ1	C-terminal GFP	*ccp2::mCherry*	CAAAAGGACAAGCACAATATG	GCTTTTCTTCCCTCTTGTTA	CATCCCTCAAAATTGAATGG	GCATTGAGCAATAAGAGGAA		
polδ2	C-terminal GFP		CATCTTTATGTTCATCAGTA	GGATATGCAAATAGGGATAT	CCTTCAATATTCAAGAAGGCG	ATGTATAAAGGAGAGACAAC		
mcm7	C-terminal GFP		GGCAAGGGTAAGAGATAGTG	CCAGTTACTTGTTCTTCTGAC	ACACGGCTATTGCTTTCCCA	CTCTAGAAACCTCATTAGTGTC		
rpa1	C-terminal GFP		GGATTTCTCTGGTTCAATAAC	GCACACATACATACAGTATTG	GAGATCCCTGAACGACAATTC	TACCGCTTTGGCAACTTTCA		

Oligo sequence for gene knockout plasmid construction

Gene name	KO	Gene size (bp)/deleted gene size (bp)	Left homologous arm		Right homologous arm		Target site of sgRNA	
			Forward primer	Reverse primer	Forward primer	Reverse primer	Oligo (Forward)	Oligo (Reverse)
ap2-o3	full length CDS	2316/2313	CCCGGTACCCGCTTATTTATTCGCTTAAT	GGGCCATGGTCTTTGAAGCGGTGTAAAA	GGGCTCGAGTAGTGTCTCCAATGATGGCTG	CCCGAATTCACACATTCACATGTGATTAT	TATTGGGCATACAAATTACAAAAG	AAACCTTTTGTAATTTGTATGCCC

Primers of diagnostic PCR for gene knockout

Gene name	KO	P9	P10	P11	P12	P13	P14	
ap2-o3	full length CDS	TATTCGCTTAATTATTCGC	GGGAATATGAAACTTAGTAGTG	ACATTTGATATTCATGCAAGAC	GCCAAATTAATAGGAAAACGA	GAGTTCTTAGAGATTAATC	GTAACTGACGGTATTTCAG	

Oligo sequences for ap2-o3 promoter swap plasmid construction

swaped promote	parental strain	Left homologous arm		Right homologous arm		Target site of sgRNA		
		Forward primer	Rerverse primer	Forward primer	Reverse primer	Oligo (Forward)	Oligo (Reverse)	
ccp2 5'utr	*ap2-o3::6HA*	ACGAAGCTTCACAAAATAAAC	GGGCCATGGCATCATTTTGATGAATATTATTC	GGGCTCGAGATGAATACACAATTTGAAA	CCCGAATTCCATTGATCATTTATTTGTTC	TATTGTGGAATTTCATGTTAAATG	AAACCATTTAACATGAAATTCCAC	

Primers for PCR-genotyping with ccp2 promoter swap

swaped promote	parental strain	P15	P16	P17	P18			
ccp2 5'utr	*ap2-o3::6HA*	GCTTCACAAAATAAACCAACGA	CTTACATAATTCTTTAAATCTAG	CATTATATATATTTAACGGAATA	GTATCATTTGTGTTATCTTTAT			

Primers for ectopic expression in p230p locus

gene of interest	parental strain	P19	P20	P21	P22	P23	P24	
mCherry	*dhc1::gfp*	GGAAAAGTATGATAACGATG	GGTTTTAATTCATTTGGATC	TAGGAACACACACACATATTAGT	GGAAATTACAAAGAAGTTAC	ATGTTGTCTCTTCAATGATT	AGATGATATCGCTATATATC	
ap2-o3::6HA	*dhc1::gfp*							

Primers for qRT-PCR

Gene name	Gene ID	Forward Primer	Reverse Primer		Gene name	Gene ID	Forward Primer	Reverse Primer
ap2-o3	PY17X_1017000	AAATGTTTGCCCAGAGTTTCAG	TTTGCTCCTTCGGACCATC		*RNASEH2A*	PY17X_1124200	GTGCTGCATCAATATGTGCTAAA	CCTGGATATCCTGATCCAAATCC
ap2-o	PY17X_0905900	TGGCTAGTCAATTGGGTACTGA	TCCAGAAGTGAGTACCCATTTTG		*POLE1*	PY17X_1130600	TGGCGTTACAAACTAGGACAA	CCCTCCTTCTCACCCTTATCTA
RFC2	PY17X_0315200	TGCATCTGATGATCGTGGTATT	CATGGTGGCAATGGTTCATTAT		*PCNA*	PY17X_1139300	GTCGAATTAAGCAGTAAAGAGCTAAC	GCAACTTCTGCATCTCCTACTAA
RFC1	PY17X_0316700	GAAATGGCAACAGGAGGTTATTC	ATCACCACTAGACATTCCATCAA		*FEN1*	PY17X_1209600	GTGAACTACAACCTATCGACTACAA	TCGCTTAATCCATCAGAAGAACT
pol δ2	PY17X_0408600	GAAGATGAAACAGCACGGTTAAA	GAACCATTGCTCATACCAACAC		*XPA*	PY17X_1224200	GGACACAACAAATTCGATTAC	AAATAGATCGACAATCTGAGC
RPA1	PY17X_0419400	CTGATGTACATGGTGATTCGATTTC	GCAATCGAAACTCTTCCTTTGG		*RFC3*	PY17X_1330600	GATTGATGTTAAACCCTCCA	AGTGTGGCAGTTCACCTTT
pol δ1	PY17X_0502300	CACGTAAACGAATTGCCACTTAT	TGTACCTGTAACTCTAGCCATTTC		*CCNH*	PY17X_1331800	TGAGCATACCTAAATACGCTGAT	CATTCCACTATTTGAGGCTGTAATC
pola1	PY17X_0615900	GAAGTAGTCGACGAAGATGG	GGTGATCATGCTATAGTCAACCG		*MPG*	PY17X_1335100	ACCAATGTTTTTGAATGGTGGT	AAATAGCATCTGGGACATTTTC
mcm7	PY17X_0805800	TAATGGGAGATCCTGGAGTAGC	CCAACCGAACTACTACCCTTTC		*APEX*	PY17X_1352400	CCAGTTATTGTCGCTGCTTTG	ATTGTTTGATGCCTGTGTGAAA
mutY	PY17X_0920400	TCTCAACATTGTGAAGAGGTAAGT	GGCATATTGAACATTGAGGAGATG		*TF* II H2	PY17X_1415100	GAACGGGATTTCAAACCAAACA	ATGCTACTACTCCAACATGCC
RFC3	PY17X_0938500	CCGAGAAGCCTTGACGAATTA	CCCACCTGGTGATCCATAAA		*MCM4*	PY17X_1417300	CCAGTATCTAGTAGGCGAACAAAG	GCTAAGGGTGTGTGACCATATC
CETN2	PY17X_0943900	GCATGTTCTCTTTGTTTGATCCT	GCTCAGATTCTTCAATTTCTTCCC		*PCNA*	PY17X_1443900	ATGGATTGCAGTCATGTATC	TCAGTACTAGATTGTTCTTCTTC
FEN1	PY17X_1007500	CAATTGACGCATCCATGTCTTT	TGAAGTCGTTTCACCTGACTC		*RFC2*	PY17X_1457600	GGGAATATGCCTAATTTGCT	GCTTGACTCCCTAACATTTC
MSH2	PY17X_1018600	CAGGACCAAATATGGGAGGAAA	GATGCACCTACTCGACACATAA		*TF* II H4	PY17X_1460000	CCCATAGTTATAGTGGTAGTTATGCT	GTGATCCACCCACTTCAGTT
DOZI	PY17X_1220900	CAGATATTAATGTATTCAGC	GGGTAATACCTTTTAATGTT		*CITH*	PY17X_1304900	CTGCAAATAGTAACATACAG	CCTTGTTGTTTATCTAATGT
P25	PY17X_0516000	CTATAATGCAGCAATTACAC	CATTAGACACATGCACAAAG		*P28*	PY17X_0515900	CCAACTTGCGATAAGATATA	CCATATCCAGGAATACATTT
GAP45	PY17X_1440100	CTACCACTCCATGTGATATG	GTATCGGATAAATCAATCT		*GAP40*	PY17X_1116500	GTTGGTTTGACATTATGTA	CACCACACACATAGGCAACT
IMC1i	PY17X_0707400	ATGACATCTCAAATGGAAA	GTTACAGGTACTTTAACAAC		*IMC1c*	PY17X_1205100	GATAACATAGACACTAAAG	ACTGGAACTTCAACAGTCTT
GAPM1	PY17X_1343600	GAGCACTACCAGGAACTGTT	CTTCGGTCAATTGTAAGAA		*GAPM2*	PY17X_0525300	CCCCAAGTTCAGTATCCATC	ACCCTGTTCTTAATAAATGC
conserved protein	PY17X_1322400	GCAACAATTTCAAATGTATG	GACATATACAGGTGGTAATG		*conserved protein, unknown function*	PY17X_1359200	GAACTTGCCAATCTAACAA	GACTGGTCATAAAGCCCAT

Primers for ChIP-qPCR

Gene name	Gene ID	Forward Primer	Reverse Primer		Gene name	Gene ID	Forward Primer	Reverse Primer
pol δ2-positve	PY17X_0408600	CAAACAAACGGAAAAAGATA	GATGTTACCTTTTCTCACAT		*RPA1-positive*	PY17X_0419400	CGCAAAATAATCCAAATGAA	ATTGCCCCCAATTATATGAGA
pol δ2-negative	PY17X_0408600	GAAACCAACCGTCAAATTAG	CACATGTGCTTGTAGCAATG		*RPA1-negative*	PY17X_0419400	GCTACTTGTCTTTCTCATCA	CTAAGGAACTTATTATAGAG
pol δ1-positive	PY17X_0502300	ATGCATATGCCCACACGGTG	CACTTAAAGGTTTTAATGGAA		*mcm7-positive*	PY17X_0805800	ATGGCCCGAATTGAAATGTG	GAGAAATCGCTGAAAAGCAG
pol δ1-negative	PY17X_0502300	GTCCTGGCAATTAAATTATA	GTGTATTTTGTGTACACATG		*mcm7-negative*	PY17X_0805800	CACACACCTTTTGTTTCGTG	CACACATGAATATTACATCC

PCR Peimers for sequence amplification

product	application	parental strain	Primers					
			Forward	Reverse				
mScarlet	*ap2-o3::mScarlet*	17XNL	GGGCCATGGtaGCCGGTTCTGCTGCTAGAGTGAGCAAGGGCGAGGCAGT	GGGCTCGAGTTACTTGTACAGCTCGTCCATGC				
mCherry	ectopic expresssion in p230p locus	*dhc1::gfp*	GGGCCATGGTGAGCAAGGGCGAGGAGGAT	CCCGAATTCCTACTTGTACAGCTCGTCCA				
migs 5'utr	ectopic expresssion in p230p locus	*dhc1::gfp*	CCCGGATCCGTCGCTACTAATTTTCGCTC	CCCGCTAGCTTTGTATATTGCTTATTCAG				
ap2-o3::6HA	ectopic expresssion in p230p locus	*dhc1::gfp*	CCCGCTAGCATGAATACACAATTTGAAAA	GGGCTCGAGTGATCGTTCCACTTTTTAGC				
dhfr 3'utr	ectopic expresssion in p230p locus	*dhc1::gfp*	GGGCTCGAGGATCCCGTTTTTCTTACTTATA	GGGCTCGAGCCGAAATTGAAGGAAAAAAC				
Py ap2-o3	*P.yoelii ap2-o3* endogenous complementation	Δ*ap2-o3*	GGGCCATGGCGATGAATACACAATTTGAAAATT	CCCGCTAGCATATTTTTTTGGTCGCTGTAAA				
Pfap2-o3	*P.falciparum ap2-o3* endogenous complementation	Δ*ap2-o3*	GGGCCATGGCGATGACACAGTATTTTGAAAATT	CCCGCTAGCATTATCAAGGAGATGATTATT				
ccp2 5'utr	promoter swap	*ap2-o3::6HA*	GGGCCATGGGTTAGATCATTCTATATATAG	GGGCTCGAGGTAATACATAAATATAAAATAAT				

Note: the sequences with underline are designed for the restriction enzyme digestion.
