# Supplementary material for: Plasmodium transcription repressor AP2‐O3 regulates sex‐specific identity of gene expression in female gametocytes
Source: EMBO Rep. 2021 Mar 4;22(5):e51660. doi: 10.15252/embr.202051660 (PMC8097350; doi:10.15252/embr.202051660)
Supplement: Supplementary file 2 — Expanded View Figures PDF [file EMBR-22-e51660-s002.pdf]

Expanded View Figures

Figure EV1. AP2-O3 is expressed in the female gametocyte and mature oocysts.

A IFA analysis of AP2-O3 expression in asexual blood stages (ABS), gametocytes, ookinetes, oocysts, and sporozoites of the *6HA::ap2-o3* and *ap2-o3::6HA* strains using anti-HA antibody. Hoechst 33342 (blue) is used for nuclear stain.
B Representative images of the mScarlet fluorescence protein expression in different stages of the living *ap2-o3::mScarlet* strain.
C Western blot of AP2-O3 expression in ABS, gametocytes, and ookinetes of the 17XNL and *ap2-o3::6HA* strains. BiP as loading control.
D Co-staining of AP2-O3 and α-Tubulin (male gametocyte specific) in gametocytes of *6HA::ap2-o3* and *ap2-o3::6HA* strains.
E Co-staining of AP2-O3 and mCherry in gametocytes of the *ccp2::mCherry;ap2-o3::6HA* strain. mCherry is specifically expressed in the female gametocytes.

Data information: In (D and E), x/y is the number of a cell displaying signal / the number of cells tested. Scale bars = 5 μm in all images. All experiments were repeated three times independently.

© 2021 The Authors

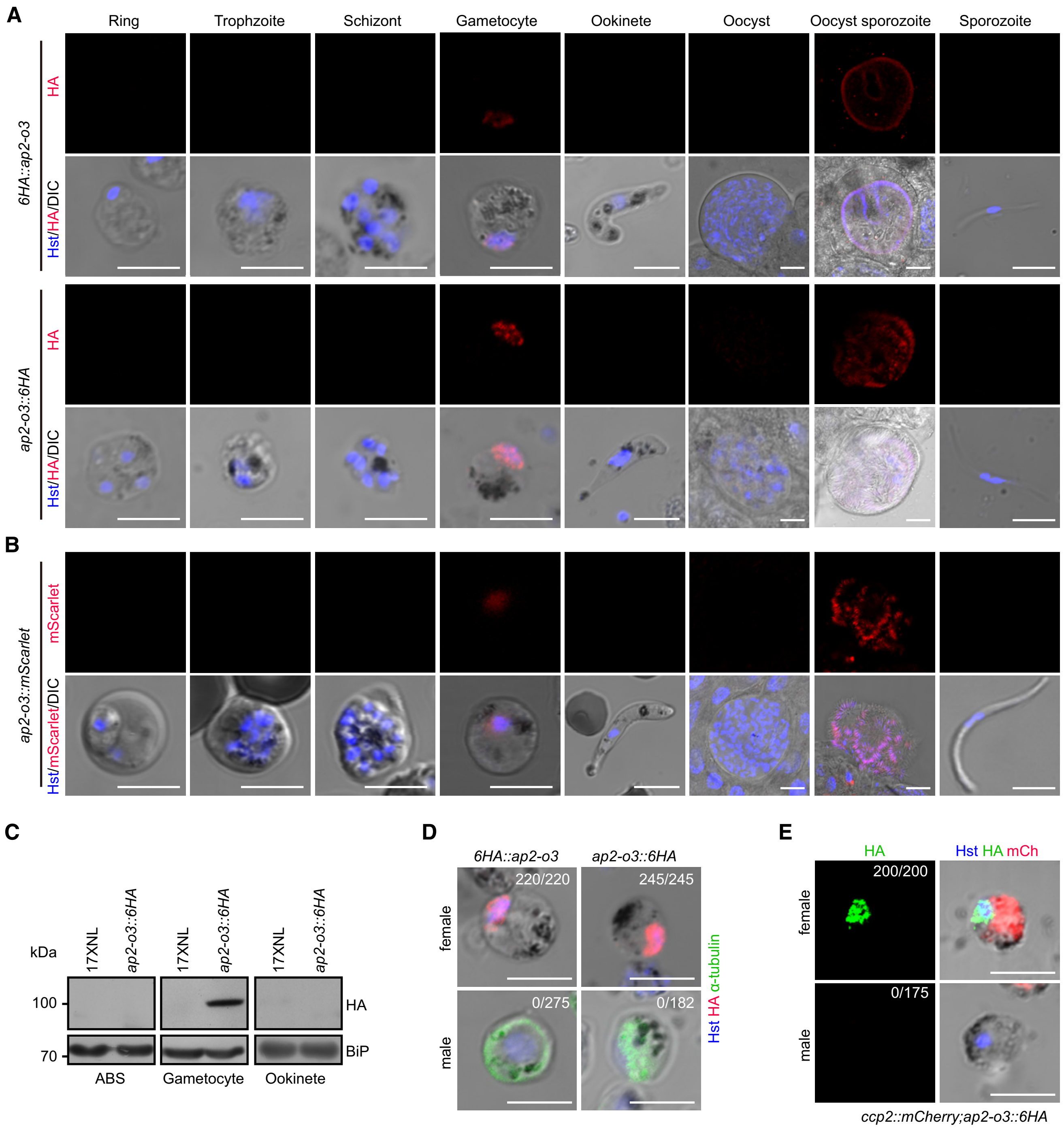

Figure EV1.

 © 2021 The Authors

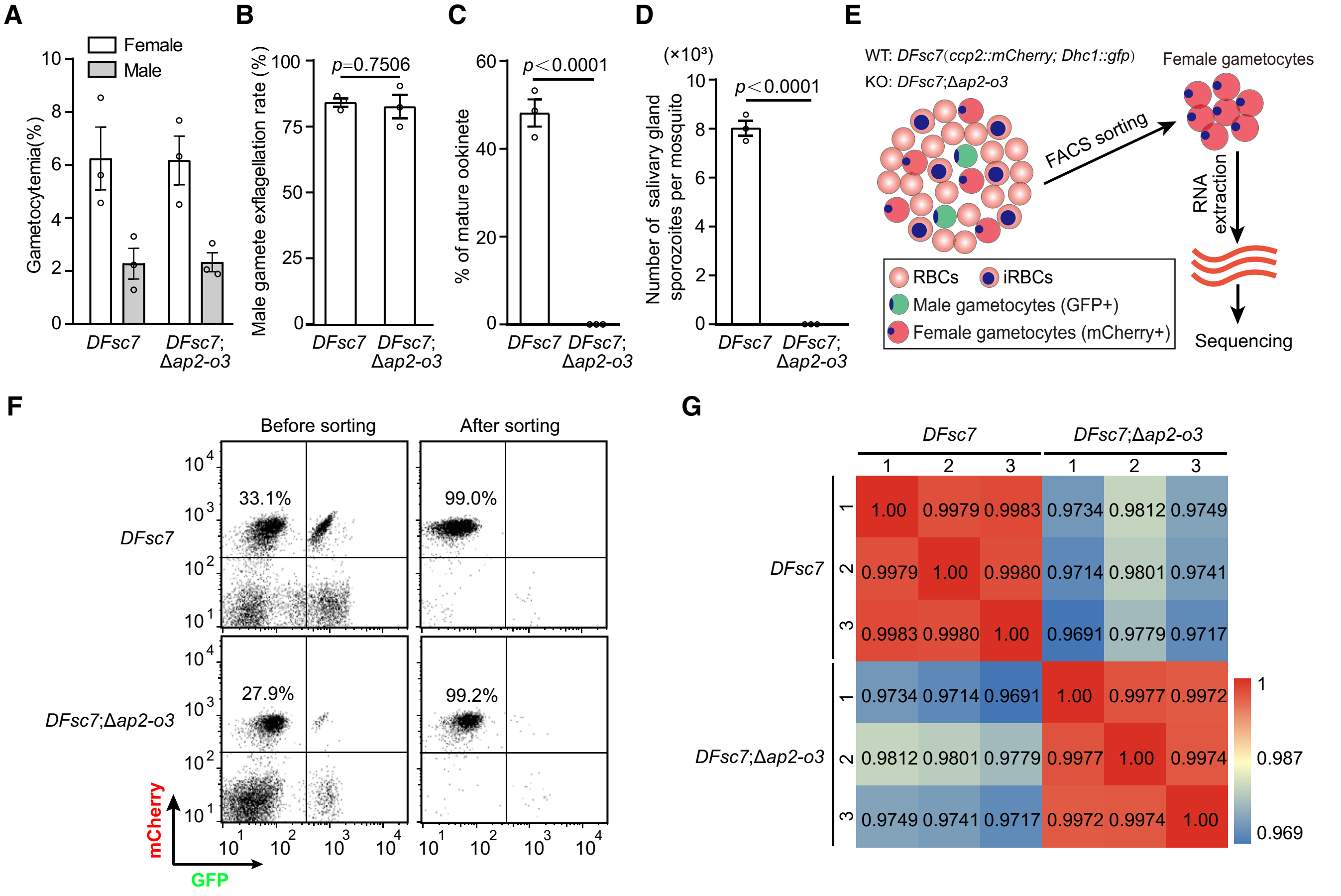

Figure EV2. Characterization of parasite strains for female gametocyte purification and transcriptome analysis.

A–D Phenotypic analysis of the *DFsc7* and *DFsc7;Δap2-o3* strains, including gametocyte (A), male gamete (B), mature ookinete (C), and salivary gland sporozoites (D). The *DFsc7* is used as a parental strain. mean ± SEM from three infected mice or three independent experiments. Two-tailed unpaired Student's *t*-test applied.

E Flow chart showing female gametocyte purification for RNA-seq.

F Flow cytometry detection of $mCherry^{+}$ (female gametocyte) and GFP^{+} (male gametocyte). The percentage numbers indicate the purity of female gametocytes.

G Correlation coefficient of global gene expression between samples of two strains. The numbers are the value of Pearson's correlation coefficient.

© 2021 The Authors

A

	Gene ID	Gene name
1	PY17X_1461900	actin I putative
2	PY17X_1104200	actin-depolymerizing factor 1 putative
3	PY17X_0420500	alpha tubulin 1
4	PY17X_0524100	alpha tubulin 2
5	PY17X_1210100	tubulin beta chain putative
6	PY17X_1230100	tubulin gamma chain putative
7	PY17X_0919000	armadillo repeat protein PF16
8	PY17X_0105700	calcium-binding protein putative
9	PY17X_1012200	calmodulin putative
10	PY17X_1311900	calmodulin-like protein
11	PY17X_0207700	centrin-1 putative
12	PY17X_1314200	centrin-2 putative
13	PY17X_0512900	centrin-3 putative
14	PY17X_0505500	conserved Plasmodium protein unknown function
15	PY17X_1126700	conserved Plasmodium protein unknown function
16	PY17X_1127300	conserved Plasmodium protein unknown function
17	PY17X_1433800	conserved Plasmodium protein unknown function
18	PY17X_1333900	dynein beta chain putative
19	PY17X_0418900	dynein heavy chain putative
20	PY17X_0508400	dynein heavy chain putative
21	PY17X_0927400	dynein heavy chain putative
22	PY17X_0826200	dynein intermediate chain putative
23	PY17X_1019800	dynein intermediate chain putative
24	PY17X_0708400	dynein light chain 1 putative
25	PY17X_1431600	dynein light chain 1 putative
26	PY17X_0935600	dynein light chain Tctex-type putative
27	PY17X_0831600	dynein light chain putative
28	PY17X_1024300	dynein light chain putative
29	PY17X_0505400	flagellar outer arm dynein-associated protein putative
30	PY17X_0836400	profilin putative
31	PY17X_0944800	radial spoke head protein putative
32	PY17X_0107700	spindle assembly abnormal protein 6 putative
33	PY17X_1329500	thioredoxin-like protein
34	PY17X_1111500	WD repeat-containing protein putative

B

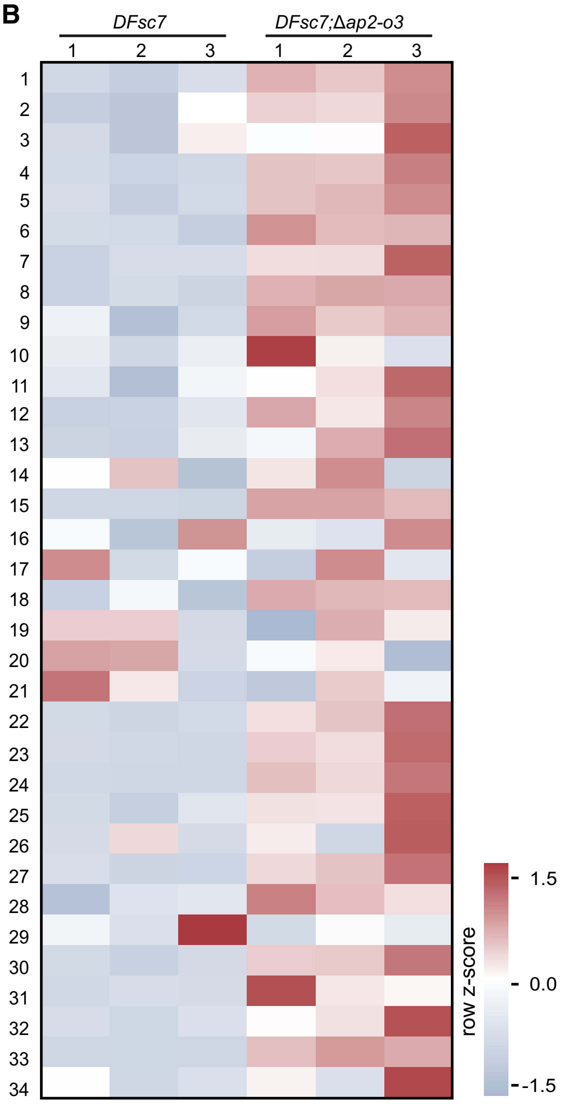

Figure EV3. Upregulation of the flagellar genes in AP2-O3 null female gametocytes.

A List of 34 male flagellar-related genes upregulated in the AP2-O3 null female gametocytes.

B Transcript expression heatmap by RNA-seq of these 34 genes between *DFsc7* (WT) and *DFsc7*;*Δap2-o3* (KO) strains. The FPKM values in each row (each gene) were normalized by Z-score normalization. The numbers from −1.5 to +1.5 are the *Z*-score value in the heatmap.

© 2021 The Authors

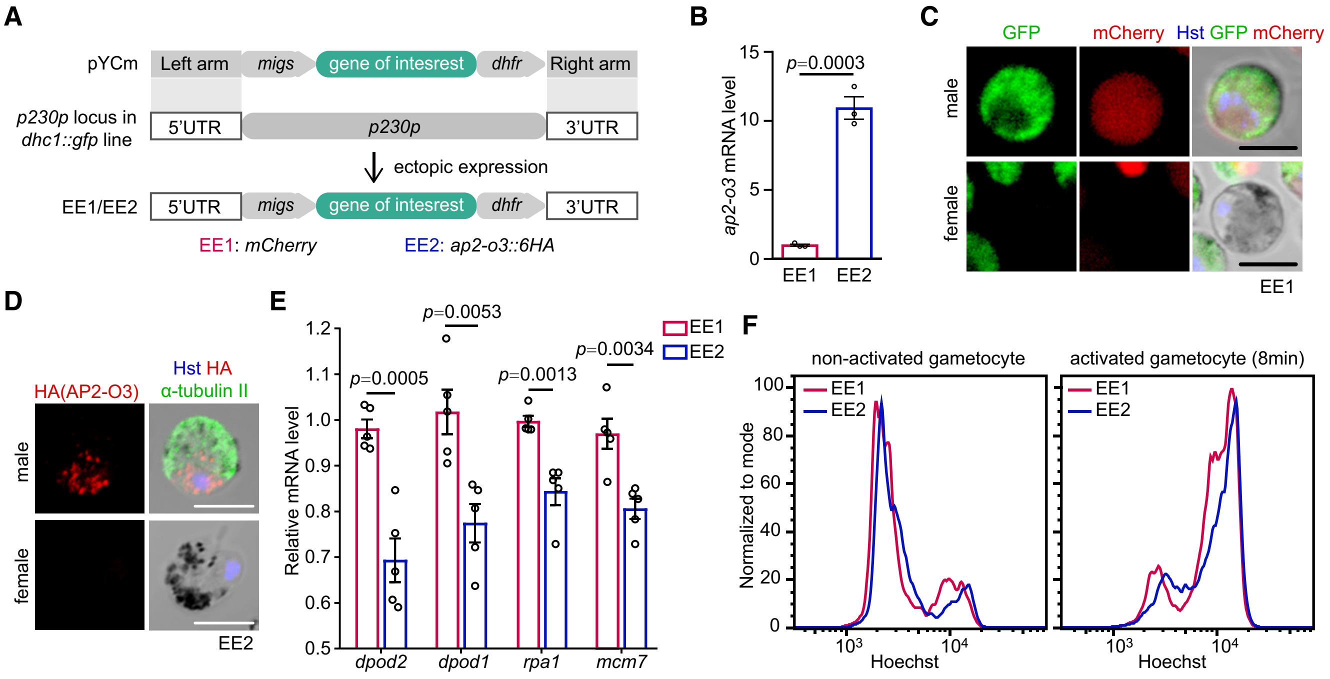

Figure EV4. AP2-O3 ectopic expression in male gametocytes inhibits transcription of male genes.

A Schematic of CRISPR/Cas9-mediated ectopic expression of the *ap2-o3* in male gametocytes. Expressing cassettes *of mCherry* (control) and *ap2-o3::6HA* under control of the 5′UTR (1,300 bp) of male gametocyte specific gene *migs* and the 3′UTR (561 bp) of *dhfr* gene were inserted into the *p230p* gene locus of the male gametocyte reporter line (*dhc1::gfp*), generating the stably transgenic lines EE1 and EE2, respectively.

B qRT–PCR of transcript level of *ap2-o3* in purified gametocytes of the EE1 and EE2. mean ± SEM from three independent experiments.

C Fluorescent microscopy detecting specific expression of mCherry in male gametocytes (GFP^{+}) of EE1.

D Co-staining of AP2-O3 and α-Tubulin (male gametocyte specific) in gametocytes of EE2.

E qRT–PCR of transcript level of male genes (*dpod2*, *dpod1*, *rpa1*, and *mcm7*) in purified gametocytes. mean ± SEM from five independent experiments.

F Flow cytometry detection of nuclear DNA (Hoechst 33342 staining) in non-activated male gametocytes and activated male gametocytes (8 min) of EE1 and EE2 lines.

Data information: Two-tailed unpaired Student's *t*-test applied in (B and E). Scale bars = 5 μm for all images.

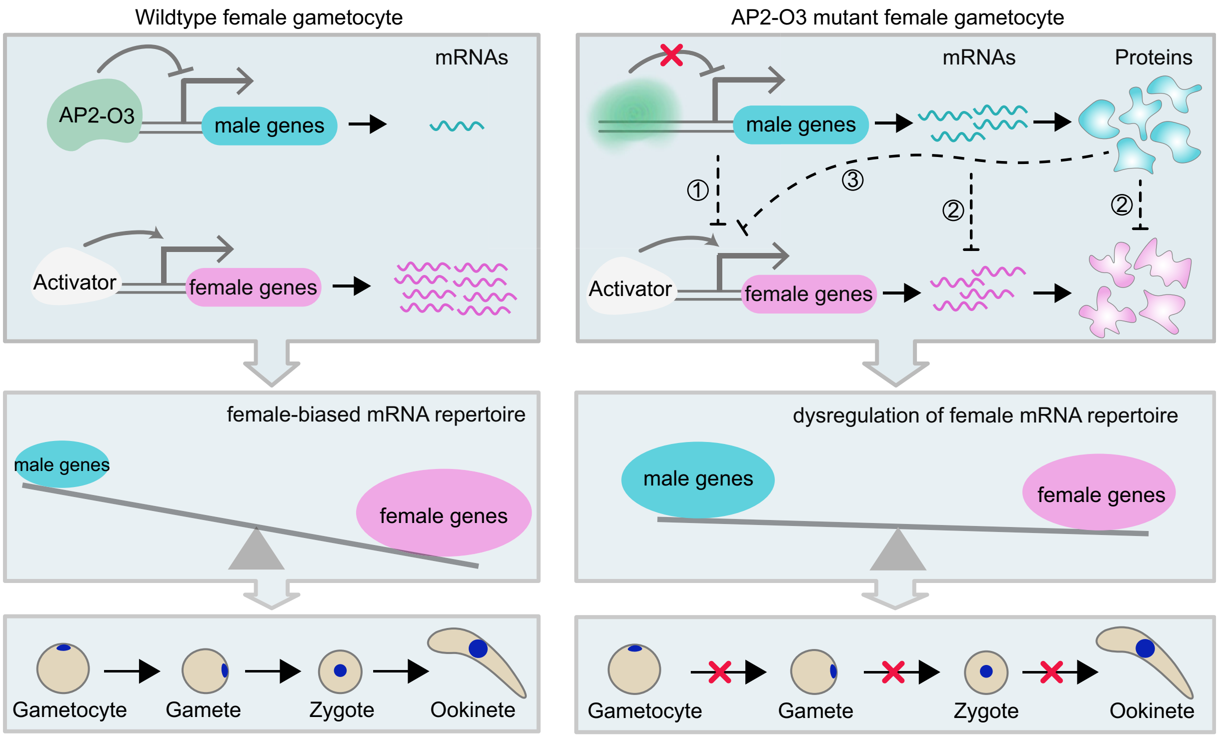

Figure EV5. Proposed model of AP2-O3 as transcription repressor in regulating the sex-specific gene expression in female gametocytes.

In female gametocytes of malaria parasite, transcription factor AP2-O3 inhibits global transcription of male (or non-female) genes to safeguard proper expression of the female genes, which is essential for the female gametogenesis, fertilization, and zygote to ookinete differentiation (left panel). Three possible mechanisms may explain the downregulation of female genes or development defect in the AP2-O3 disrupted female gametocytes (right panel). One is that there exists a competition mechanism between female and male (or non-female) gene expression program in the female gametocytes. Global upregulation of male genes expropriates the resource needed for transcription and translation of the female gene expression programs. Two is that in the absence of AP2-O3, a superabundance of male transcripts or male proteins subverts the function of female transcripts or female proteins. Three is that among the upregulated genes caused by AP2-O3 disruption, unknown factor could repress the female gene expression.

 © 2021 The Authors
